# Client Satisfaction and Experience With Telepsychiatry: Development and Validation of a Survey Using Clinical Quality Domains

**DOI:** 10.2196/19198

**Published:** 2020-09-29

**Authors:** Eva Serhal, Anne Kirvan, Marcos Sanches, Allison Crawford

**Affiliations:** 1 Virtual Mental Health and Outreach Centre for Addiction and Mental Health Toronto, ON Canada; 2 Krembil Centre for Neuroinformatics Centre for Addiction and Mental Health Toronto, ON Canada

**Keywords:** telemedicine, psychiatry, mental health, patient satisfaction, quality of health care

## Abstract

**Background:**

Telepsychiatry is an increasingly used model of mental health care that connects patients with psychiatrists at a distance via videoconference. Telepsychiatry is an effective clinical intervention that improves access to quality care in regions with limited resources or in clinical situations where in-person care is unavailable.

**Objective:**

This study aims to develop a validated survey tool to measure patient experience and satisfaction with telepsychiatry based on the quality of care domains. This study also seeks to understand which health service outcomes were most strongly correlated with overall satisfaction in the context of telepsychiatry.

**Methods:**

The survey created in this study was developed and validated with a panel of subject matter and process experts and was piloted with 274 patients who received clinical consultations through the TeleMental Health Program at the Centre for Addiction and Mental Health. Factor analysis was used to determine correlations between questions and quality of care domains and was also used to assess model fit.

**Results:**

The study provides a validated survey to measure patient satisfaction and experience with telepsychiatry across 4 domains: access and timeliness, appropriateness, effectiveness, and safety. Both safety and access and timeliness were found to be statistically significant predictors of satisfaction in our sample.

**Conclusions:**

By situating patient satisfaction and experience within this framework, the survey facilitates patient data collection and interpretation through a clinical quality lens.

## Introduction

Telepsychiatry is an increasingly common modality of mental health care that connects patients with psychiatrists at a distance via videoconference [1]. This mental health care delivery model is an effective clinical intervention that reduces geographical barriers and improves access to care in regions with limited resources [2]. Although satisfaction with telepsychiatry is commonly reported as being high among service users and service providers alike [3], opportunities for research exist in the development and validation of quantitative and qualitative indices to measure clinical service, satisfaction, and experience and thus to ensure access to quality care [4].

Patient satisfaction and experience are widely accepted as gauges of health service performance and are commonly used as measures of overall quality of care in health systems [5-9]. Keeping patient experience at the forefront of quality measurement ensures that services remain acceptable and appropriate to patients by being responsive to their needs [10]. Several patient satisfaction surveys for telepsychiatry have been developed and used in research and practice [3,11,12]. Two patient surveys that we were able to locate in the literature have been validated—one that assesses patient experiences of privacy and security [13] and another designed to measure the attitudes of laypeople and providers toward telepsychiatry [14]—yet no comprehensive satisfaction measures have been validated to date.

Validated measures of patient satisfaction have, however, been developed in the broader field of telemedicine [15,16]. Yip et al [16] identified a method of validating telemedicine tools and provided validated questions in the following domains: audiovisual quality, general satisfaction, accessibility, use of equipment and correctness of vital signs being transferred, level of comfort, and satisfaction with the telemedicine encounter.

The literature points to a number of factors to consider in the development and validation of patient experience surveys. Litwin’s [17] literature on psychometrics highlights the importance of assessing reliability criteria, selecting appropriate validity criteria, and scaling or scoring. Mazor et al [5] recommend collecting a large sample of surveys when evaluating patient experience to increase reliability of the ratings, as individual patients are more likely to provide similar ratings across items. It is also important to recognize the possibility of biases inherent to data collection caused by nonresponse. For example, satisfied patients are more likely to respond than unsatisfied patients [5,18-22].

To gain a more comprehensive understanding of patient satisfaction and experience with telepsychiatry, this research study had two primary objectives: (1) to develop a validated survey tool that measures different dimensions of client experience and satisfaction with telepsychiatry and (2) to examine the relationship between dimensions of clinical quality, as measured in the survey, and overall satisfaction.

## Methods

### Conceptual Framework

This study links several health service outcomes (HSOs) commonly used to describe patient experience and overall quality of health interventions [23-26]. The survey was developed with items relating to HSO domains to obtain a robust assessment of the overall patient experience of telepsychiatry. HSO domains included the following:

1. *Safety*: “Avoiding injuries to patients from the care that is intended to help them” [23]
2. *Effectiveness*: “Providing services based on scientific knowledge to all who could benefit and refraining from providing services to those not likely to benefit (avoiding underuse and overuse)” [23]
3. *Efficiency*: “Avoiding waste, in particular waste of equipment, supplies, ideas, and energy” [23]
4. *Access and timeliness*: Improving the “fit between the patient and the health care system” [24], and “reducing waits and sometimes harmful delays for those who receive and those who give care” [23]
5. *Appropriateness*: Perceived fit, relevance, and compatibility of the intervention [26].

Patient-centeredness, a commonly used metric of service quality, was not explicitly included as a domain in the survey, as the survey itself is intended to provide a patient-centered measure of service quality.

### Survey Development

The survey questions were generated in 2 steps. First, we identified relevant questions derived from the literature, previously developed questions drawn from historical program surveys, and any additional questions we felt were necessary to understand the quality of a patient’s experience with telepsychiatry. Next, to ensure that there was representation of questions across the HSO domains, we mapped the total set of 21 questions to the domains of safety, efficiency, access and timeliness, appropriateness, and effectiveness. We included access with timeliness because, for many patients living in rural areas, lack of timeliness is a direct result of a lack of access to local care. These domains align with other proposed domains for the measurement of telehealth, such as access, cost, experience, and effectiveness [27]. Research ethics board review was not required for survey development and use, as this is a program evaluation and quality improvement project.

### Question Validation

#### Content Validity

Following a similar validation model recommended by Yip et al [16], a panel of 7 subject matter experts were engaged, including 4 psychiatrists that deliver telepsychiatry, a social worker, a research coordinator, and an administrative director, as well as 3 process experts in survey design and marketing. This panel was asked to review the survey for relevance, clarity, plain language, and consistent distribution of questions across HSO domains. The panel was pulled together as a group to meet and discuss the questions. Those who were not able to participate in person were emailed with written instructions that asked them to do the following:

1. Rate on a scale of 1-5 how important the question is to include in the survey
2. Rate on a scale of 1-5 the suitability of each question within its HSO domain
3. Rate on a scale of 1-5 how well the survey measures each HSO domain
4. Recommend new questions if something important is missing
5. Flag awkward or unclear questions for discussion by the survey design panel.

Where there were discrepancies, there was discussion between the team members about the nature of the discrepancy until consensus was reached.

#### Testing the Validity of the Survey

This survey was piloted with patients who received clinical consultations through our telepsychiatry program. Although Yip et al [16] validated their patient survey with 38 patients, our study included a sample size of 274 patients for validation to ensure appropriate representation and sufficient power to conduct analysis of all 21 questions.

The 21-question survey was printed and sent to telemedicine coordinators from 25 referring primary care sites throughout Ontario. Site selection was based on primary care teams that regularly referred to the program and that had dedicated telemedicine coordinators to support anonymous survey collection. The sites were primarily in rural or underserved areas. Coordinators were provided with instructions that requested that they provide a survey to each telepsychiatry patient after the patient completed their telepsychiatry appointment. It is important to note, however, that 8 of the sites did not return any surveys, and there was no opportunity to determine the overall response rate.

This study used convenience sampling, a nonprobability sampling method, whereby a survey was provided to all patients who completed a telepsychiatry appointment. Random sampling was not possible based on the nature of the clinical service. Each survey had a unique identifier and a code for the primary care organization name. No personal identifiers were collected.

Each site was sent 10 paper-based surveys and 10 letter-sized envelopes, an instruction page, a schedule for collection of surveys, and an introductory letter to the site’s executive director and telemedicine coordinator. More surveys and envelopes were sent on an as-needed basis. Instructions asked the telemedicine coordinators to provide each patient, at the beginning of their telepsychiatry consultation, with a copy of the survey, an envelope, and a pen. Telemedicine coordinators then returned to collect the completed and sealed surveys at the end of the consultation. Each mailed survey package included 4 postage-paid manila envelopes for quarterly collection. Quarterly emails asking each site to send us all of their collected anonymous patient satisfaction surveys were sent as a reminder.

#### Factorial Structure

To test the structure of the scale, with previously defined 5 constructs, a confirmatory factor analysis (CFA) model was adjusted to the data using Mplus 7.11 [28]. The 5-point ordinal Likert scale was accommodated by the weighted least squares with mean and variance-adjusted chi-square statistic (WLSMV) estimator in Mplus [29]. The WLSMV estimator, which is robust to non-normality, uses polychoric correlation and provides adjusted chi-squared statistics. Simulations conducted by Flora and Curran [30] show that the WLSMV estimator is likely to work well with our sample size. The WLSMV estimator uses all available data through pair-wise correlation calculation and assumes missing completely at random. Goodness of fit for the CFA models were assessed using the chi-square statistic (for which a significant value is considered to be evidence of lack of fit), the root mean square error of approximation (RMSEA), and the confirmatory fit index (CFI). The chi-square statistic is sensitive to sample size and departures from multivariate normality, which justifies a focus also on the other fit indices [31]. All the inferential statistical analyses described in this study accounted for the clustering of patients within each of the 17 primary care sites, using an extension of the pseudo-likelihood method, described by Asparouhov and Muthem [32].

Once the initial CFA was conducted, model respecification was conducted based on the interpretation of the model according to the meaning of items, original model coefficients, and exploratory factor analysis (EFA), which used Geomin Rotation with 4 and 5 factors to be consistent with the theory underlying the development of the tool and with results from the initial CFA. The EFA was also conducted in Mplus 7.11, with the WLSMV estimator for ordinal data. A final test of the structure was conducted using CFA. Given that the respecification and test of the final model used the same data used to fit the original CFA, we recognize that replication with independent data would add evidence to our results.

#### Reliability

Once all client experience scales were obtained, their reliability was assessed. Reliability of a scale has to do with the extent to which the scale produces similar results under similar conditions and is measured via the internal consistency of the items used. This type of reliability has been popularly estimated by Cronbach alpha [33]; however, this method has been criticized [34,35]. The reported reliability estimates in this study are, therefore, calculated by the method outlined by Raykov [35] and can be interpreted in the same way as the coefficient alpha: the closer to 1 its value is, the higher the reliability, with values higher than 0.7 being considered acceptably reliable [32,36].

### Impact on Overall Satisfaction

The client satisfaction and experience survey is useful on its own by providing a tested tool that measures aspects of patient experience with telepsychiatry. Under the assumption that these experiences are important drivers of overall attitudes of patients toward health care services and institutions and that these attitudes can be important summary indicators of overall performance, we extended our final CFA model by specifying causal paths from the validated experiences to the overall satisfaction, also included in our questionnaire as a 5-point Likert scale question. As this is just an extension of our final CFA, its technical details are the same as described above for the CFA model.

## Results

### Descriptive Analysis

Of the 274 patients who returned the questionnaire in the 2016/2017 and 2017/2018 fiscal years, 147 (53.6%) reported their gender as female, 118 (43.1%) reported their gender as male, 3 reported their gender as transgender, and 1 reported their gender as intersex. Moreover, 4 participants reported their gender as other and 3 preferred not to answer. With respect to age, 25.5% of the participants were aged less than 30 years, 36.2% were between 30 and 49 years, 36.9% were above 50 years, and 5 respondents reported that they preferred not to answer. A total of 175 patients (63.9%) stated this to be their first experience with telepsychiatry, and 26 (6.5%) patients had been hospitalized for mental health issues in the previous 12 months. At the end of the study, 274 surveys were completed in 17 different primary care sites. Items missing values were observed in 57 surveys, 72% of which had 3 or fewer missing values.

In general, patient-reported experiences tended to be very positive; 58% of the patients strongly agree with the overall satisfaction question and 91% either strongly agree or agree. The complete results for all survey questions are shown in Table 1.

**Table 1:** Client satisfaction survey results.

Questions	Number of survey responses, n	Strongly disagree, n (%)	Disagree, n (%)	Neutral, n (%)	Agree, n (%)	Strongly agree, n (%)
Q1. I am satisfied with the length of time I had to wait between my referral and the Telepsychiatry appointment.	269	5 (1.9)	27 (10.0)	40 (14.9)	107 (39.8)	90 (33.5)
Q2. It was easy to book my Telepsychiatry appointment.	267	3 (1.1)	11 (4.1)	26 (9.7)	116 (43.4)	111 (41.6)
Q3. During my Telepsychiatry appointment, I was able to see the psychiatrist clearly.	270	6 (2.2)	1 (0.4)	12 (4.4)	79 (29.3)	172 (63.7)
Q4. During my Telepsychiatry appointment, I was able to hear the psychiatrist clearly.	271	3 (1.1)	4 (1.5)	13 (4.8)	81 (29.9)	170 (62.7)
Q5. I am confident that the psychiatrist and my health care providers are working as a team.	270	4 (1.5)	1 (0.4)	17 (6.3)	100 (37.0)	148 (54.8)
Q6. I feel that there was an adequate amount of time allotted for the Telepsychiatry appointment.	270	5 (1.9)	8 (3.0)	14 (5.2)	97 (35.9)	146 (54.1)
Q7. I felt comfortable during my Telepsychiatry appointment.	270	5 (1.9)	5 (1.9)	45 (16.7)	96 (35.6)	119 (44.1)
Q8. I believe Telepsychiatry is just as effective as an in-person psychiatry appointment.	269	11 (4.1)	20 (7.4)	55 (20.4)	86 (32.0)	97 (36.1)
Q9. I was able to get an appointment through Telepsychiatry sooner than an in-person psychiatry appointment.	257	7 (2.7)	10 (3.9)	51 (19.8)	84 (32.7)	105 (40.9)
Q10. I felt that confidentiality was protected throughout my Telepsychiatry appointment.	268	4 (1.50)	2 (0.7)	10 (3.7)	107 (39.9)	145 (54.1)
Q11. The psychiatrist understood my concerns.	266	6 (2.3)	1 (0.4)	18 (6.8)	102 (38.3)	139 (52.3)
Q12. The psychiatrist treated me with courtesy and respect.	269	4 (1.5)	1 (0.4)	4 (1.5)	62 (23.0)	198 (73.6)
Q13. The psychiatrist explained my diagnosis in a way that I could understand.	259	4 (1.5)	2 (0.8)	31 (12.0)	87 (33.6)	135 (52.1)
Q14. The psychiatrist involved me in decisions about my treatment plan.	260	3 (1.2)	1 (0.4)	33 (12.7)	100 (38.5)	123 (47.3)
Q15. The psychiatrist explained the benefits and risks of any medications he/she recommended.	254	2 (0.8)	11 (4.3)	48 (18.9)	81 (31.9)	112 (44.1)
Q16. I am confident that I will be able to follow the psychiatrist’s recommendations.	264	4 (1.5)	5 (1.9)	43 (16.3)	93 (35.2)	119 (45.1)
Q17. I understand what to do if I have a mental health emergency following this appointment.	265	4 (1.5)	14 (5.3)	32 (12.1)	98 (37.0)	117 (44.2)
Q18. The physical location of my Telepsychiatry appointment was convenient for me to get to.	269	4 (1.5)	5 (1.9)	11 (4.1)	90 (33.5)	159 (59.1)
Q19. I experienced a significant improvement in my mental health while I was waiting for my Telepsychiatry appointment.	260	54 (20.8)	71 (27.3)	79 (30.4)	27 (10.4)	29 (11.2)
Q20. I experienced a significant decline in my mental health while I was waiting for my Telepsychiatry appointment.	253	40 (15.8)	54 (21.3)	108 (42.7)	31 (12.3)	20 (7.9)
Q21. Overall, I am satisfied with the Telepsychiatry appointment.	266	5 (1.9)	1 (0.4)	18 (6.8)	88 (33.1)	154 (57.9)

### Preliminary CFA

The initial CFA tested the theoretical factor structure defined in the earlier stages of the development of the scales. The model fit was fairly good, but it did indicate some inconsistencies with the factor structure as defined (how the questions related to the HSO domains). The chi-square was 373.34 with 160 degrees of freedom and *P*<.001. The RMSEA (0.07) and CFI (0.97) both indicated that the proposed model was acceptable overall; however, the factor loadings demonstrated that several of the questions did not align well with other factor items, as indicated by coefficient variance and nonsignificance, particularly with respect to effectiveness and efficiency (Table 2).

**Table 2:** Factor coefficients for the preliminary confirmatory factor analysis.

Survey questions and factors		Estimate^a^	SE^b^	Estimate/SE^c^	*P* value^d^
**Factor 1: Access and timeliness**					
	Q1. I am satisfied with the length of time I had to wait between my referral and the Telepsychiatry appointment.	1.00^e^	N/A^f^	N/A	N/A
	Q2. It was easy to book my Telepsychiatry appointment.	1.09	0.10	11.30	<.001
	Q16. I am confident that I will be able to follow the psychiatrist’s recommendations.	1.45	0.11	13.70	<.001
	Q18. The physical location of my Telepsychiatry appointment was convenient for me to get to.	1.28	0.08	15.27	<.001
**Factor 2: Appropriateness**					
	Q8. I believe Telepsychiatry is just as effective as an in-person psychiatry appointment.	1.00^e^	N/A	N/A	N/A
	Q11. The psychiatrist understood my concerns.	1.32	0.05	25.02	<.001
	Q14. The psychiatrist involved me in decisions about my treatment plan.	1.28	0.05	27.16	<.001
**Factor 3: Effectiveness**					
	Q3. During my Telepsychiatry appointment, I was able to see the psychiatrist clearly.	1.00^e^	N/A	N/A	N/A
	Q4. During my Telepsychiatry appointment, I was able to hear the psychiatrist clearly.	1.01	0.06	16.82	<.001
	Q5. I am confident that the psychiatrist and my health care providers are working as a team.	1.18	0.03	38.14	<.001
	Q13. The psychiatrist explained my diagnosis in a way that I could understand.	1.30	0.05	25.58	<.001
	Q19. I experienced a significant improvement in my mental health while I was waiting for my Telepsychiatry appointment.	0.36	0.10	3.70	<.001
	Q20. I experienced a significant decline in my mental health while I was waiting for my Telepsychiatry appointment.	−0.01	0.06	−0.24	.81
**Factor 4: Efficiency**					
	Q6. I feel that there was an adequate amount of time allotted for the Telepsychiatry appointment.	1.00^e^	N/A	N/A	N/A
	Q9. I was able to get an appointment through Telepsychiatry sooner than an in-person psychiatry appointment.	0.70	0.05	13.03	<.001
**Factor 5: Safety**					
	Q7. I felt comfortable during my Telepsychiatry appointment.	1.00^e^	N/A	N/A	N/A
	Q10. I felt that confidentiality was protected throughout my Telepsychiatry appointment.	1.10	0.05	23.60	<.001
	Q12. The psychiatrist treated me with courtesy and respect.	1.19	0.04	27.43	<.001
	Q15. The psychiatrist explained the benefits and risks of any medications he/she recommended.	0.97	0.05	20.22	<.001
	Q17. I understand what to do if I have a mental health emergency following this appointment.	1.02	0.06	16.52	<.001

^a^Estimate, model estimate of factor loadings.

^b^SE: SE of estimates.

^c^Estimate/SE: ratio between estimates and their SE (under the assumption of normality of estimates, this ratio has a standard normal distribution, which is used to calculate the *P* value).

^d^*P* value: estimate of the probability of a coefficient equal or larger than that found under the null hypothesis.

^e^Coefficients were fixed at 1.00 to allow model identification.

^f^N/A: not appliable.

### Model Respecification

Besides not showing very good fit indices, the CFA showed some other issues that led us to respecify the model. This respecification took into account the correction of the issues in the initial model, the theoretical basis for the constructs, the modification indices from the CFA, and insights from an EFA. We detail the changes made to the model in the following section.

The efficiency factor showed different signs of poor fit. It had inconsistent correlations above 1 with other factors. Items Q6 (“I feel that there was an adequate amount of time allotted for the telepsychiatry appointment”) and Q9 (“I was able to get an appointment through telepsychiatry sooner than an in-person psychiatry appointment”) showed poor variance. On the basis of the reinterpretation of these items and the fact that they did not come together in the EFA, Q6 was moved to factor effectiveness and Q9 to factor access and timeliness. The efficiency factor was removed, as none of the questions lined up well with this domain.

Items Q19 (“I experienced a significant improvement in my mental health while I was waiting for my telepsychiatry appointment”) and Q20 (“I experienced a significant decline in my mental health while I was waiting for my telepsychiatry appointment”) in the effectiveness factor did not fit well, as can be seen in Table 2. They had almost none of their variance explained, and the EFA showed them together, but with low loadings and separated from other items. Upon reviewing the interpretation of these items, it was decided that they should be removed from the analysis because they represented an effect of the treatment received while awaiting a telepsychiatry consultation rather than an experience with the telepsychiatry service itself. Item Q16 (“I am confident that I will be able to follow the psychiatrist’s recommendations”) was moved from the access and timeliness domain to the appropriateness domain, and item Q13 (“The psychiatrist explained my diagnosis in a way that I could understand”) was moved from the effectiveness domain to the safety domain as first suggested by the largest modification indices and confirmed by the EFA and theory-related considerations. Despite other significant modification indices and some differences in the EFA structure, we did not make any further changes to the model as they were not warranted by theoretical considerations.

### Final CFA

A second CFA was run to test the structure of the revised model, using the same measures of fit as outlined above. Chi-square was 288.19 with 129 degrees of freedom and *P*<.001. The RMSEA (0.07) and CFI (0.98) continued to suggest a good fit. Overall, we observed remarkable improvement in the model fit. The reliability was lower but still acceptable for access and timeliness (0.72) and good for the other 3 factors (appropriateness=0.81, effectiveness=0.83, and safety=0.86). The final model is shown in Table 3.

**Table 3:** Factor coefficients for the final confirmatory factor analysis.

Survey questions and factors		Estimate^a^	SE^b^	Estimate/SE^c^	*P* value^d^
**Factor 1: Access and timeliness**					
	Q1. I am satisfied with the length of time I had to wait between my referral and the Telepsychiatry appointment.	1.00^e^	N/A^f^	N/A	N/A
	Q2. It was easy to book my Telepsychiatry appointment.	1.10	0.10	11.23	<.001
	Q9. I was able to get an appointment through Telepsychiatry sooner than an in-person psychiatry appointment.	0.98	0.08	11.90	<.001
	Q18. The physical location of my Telepsychiatry appointment was convenient for me to get to.	1.29	0.08	15.63	<.001
**Factor 2: Appropriateness**					
	Q8. I believe Telepsychiatry is just as effective as an in-person psychiatry appointment.	1.00^e^	N/A	N/A	N/A
	Q11. The psychiatrist understood my concerns.	1.31	0.05	24.56	<.001
	Q14. The psychiatrist involved me in decisions about my treatment plan.	1.27	0.05	27.54	<.001
	Q16. I am confident that I will be able to follow the psychiatrist’s recommendations.	1.18	0.05	22.10	<.001
**Factor 3: Effectiveness**					
	Q3. During my Telepsychiatry appointment, I was able to see the psychiatrist clearly.	1.00^e^	N/A	N/A	N/A
	Q4. During my Telepsychiatry appointment, I was able to hear the psychiatrist clearly.	1.01	0.06	16.60	<.001
	Q5. I am confident that the psychiatrist and my health care providers are working as a team.	1.17	0.03	36.97	<.001
	Q6. I feel that there was an adequate amount of time allotted for the Telepsychiatry appointment.	1.09	0.06	18.63	<.001
**Factor 4: Safety**					
	Q7. I felt comfortable during my Telepsychiatry appointment.	1.00^e^	N/A	N/A	N/A
	Q10. I felt that confidentiality was protected throughout my Telepsychiatry appointment.	1.11	0.05	23.50	<.001
	Q12. The psychiatrist treated me with courtesy and respect.	1.20	0.04	28.15	<.001
	Q13. The psychiatrist explained my diagnosis in a way that I could understand.	1.20	0.03	40.02	<.001
	Q15. The psychiatrist explained the benefits and risks of any medications he/she recommended.	0.97	0.05	19.94	<.001
	Q17. I understand what to do if I have a mental health emergency following this appointment.	1.02	0.06	16.68	<.001

^a^Estimate: model estimate of factor loadings.

^b^SE: SE of estimates.

^c^Estimate/SE: ratio between estimates and their SE (under the assumption of normality of estimates, this ratio has *a standard normal distribution, which is used to calculate the P* value).

^d^*P* value: estimate of the probability of a coefficient equal or larger than that found under the null hypothesis.

^e^Coefficients were fixed at 1.00 to allow model identification.

^f^N/A: not appliable.

### Patient Experience

Overall, the client satisfaction surveys demonstrated high ratings from patients across the 4 domains of access and timeliness (mean 16.7 out of 20, SD 2.8), appropriateness (mean 16.8 out of 20, SD 3.0), effectiveness (mean 17.9 out of 20, SD 2.6), and safety (mean 25.8 out of 30, SD 4.0), with the highest overall score for effectiveness (Table 4). The total factor score was calculated for each factor by summing the scores for each item. Subjects with missing values in at least one item of a factor did not have the total score calculated for that factor. We observe that for all factors, our sample shows total scores that are concentrated on the higher end of the scale, as can be seen in Table 4.

**Table 4:** Survey results stratified by factor.

Factor	Participants, n	Mean (SD)	SE	Minimum	First quartile	Median	Third quartile	Maximum
Access and timeliness	246	16.7 (2.8)	0.18	4	15	17	19	20
Appropriateness	252	16.8 (3.0)	0.19	4	15	17	20	20
Effectiveness	268	17.9 (2.6)	0.16	4	16	19	20	20
Safety	245	25.8 (4.0)	0.26	6	23	26	29	30

Table 5 demonstrates the coefficient of the factors as predictors of overall satisfaction. Access and timeliness, and safety were found to be statistically significant predictors of overall satisfaction based on the *P* value, appropriateness was almost significant, and effectiveness was not found to significantly predict overall satisfaction.

**Table 5:** Confirmatory factor analysis with overall satisfaction.

Factor	Estimate	SE	Estimate/SE^a^	*P* value
Access and timeliness	0.29	0.12	2.46	.01
Appropriateness	0.37	0.23	1.61	.11
Effectiveness	−0.02	0.10	−0.17	.86
Safety	0.62	0.21	2.93	.003

^a^Estimate/SE: ratio between estimates and their SE (under the assumption of normality of estimates, this ratio has a standard normal distribution, which is used to calculate the *P* value)

## Discussion

### Principal Findings

This study provides a validated survey tool to measure patient satisfaction and experience with telepsychiatry across 4 HSO domains: access and timeliness, appropriateness, effectiveness, and safety. This validated survey tool serves as a good model for future research and program evaluation that ensures patient experience is collected through a quality of care lens. By clustering items into HSO domains, survey results allow for targeted quality improvement efforts that address patient-identified gaps in service quality. This survey has global relevance in telepsychiatry as well as the broader fields of telemental health and telemedicine.

Consistent with previous research studies [3,11,12], patients’ responses suggest high levels of satisfaction with telepsychiatry services. In this study, patient satisfaction was high across all 4 domains, with the highest overall score for the effectiveness domain, followed by safety, appropriateness, and access and timeliness. Our study also sought to understand which HSOs were most strongly correlated with overall satisfaction in the context of telepsychiatry and found access and timeliness, and safety to be statistically significant predictors. The term safety in this case represents both physical and psychological safety, which is evident in the way that the six individual items clustered together around issues of effective communication, comfort, confidentiality, respect, and patient knowledge about what steps to take in case of an emergency.

Although the effectiveness of telepsychiatry is well established in the literature [37] and is a key dimension of overall quality of care [23,25], it is important to note that effectiveness was not found to be a statistically significant predictor of patient satisfaction in this study. Appropriateness was found to be almost significant; however, only access and timeliness, and safety were found to statistically significantly predict satisfaction. A potential reason for this is that the majority of patients accessing telepsychiatry in Ontario are in rural areas and thus are likely to have fairly limited access to timely service locally, so timeliness and access are major components in their overall satisfaction [38]. This information has important clinical practice and policy implications when we consider the crucial role that access, timeliness, and safety play in shaping overall patient satisfaction with telepsychiatry services, including ensuring safe spaces for patients, safety protocols and guidelines for clinicians, and a focus on timely service that supports patients with significant barriers to access.

Another possible explanation for the lack of significance of effectiveness on satisfaction may be related to the temporal relationship of the survey with the appointment, especially given that a majority of the respondents (63.9%) were receiving telepsychiatry for the first time. In other words, judgments of effectiveness may not be as salient to satisfaction immediately following a first appointment. In the future, examining survey responses after repeat appointments will be an important direction.

Although telepsychiatry programs have increased the reach of mental health services, research suggests that telepsychiatry is still underused by patients and providers alike [38]. As work on this survey neared completion, we encountered an unexpected rapid increase in telepsychiatry use in the context of the pandemic caused by COVID-19. Many commentators predict a sustained increase in the use of telehealth and other digital health technologies [39,40]. Now more than ever, understanding the perspectives of end users and stakeholders, including patients and providers, is crucial in service planning, evaluation, and research. Patient satisfaction and experience surveys provide a useful avenue for feedback that can be used to guide patient-centered quality improvement initiatives that are responsive to the needs of patients [10].

To ensure the acceptability of telepsychiatry services, it will be important to ensure that services address those factors that have been identified as the most significant contributors to overall satisfaction for patients, namely, access and timeliness, and safety. As telepsychiatry services continue to increase in use, we need to keep patient experiences and perspectives at the forefront of not only quality measurement but also program and systems planning.

### Future Directions

With a survey tool now developed and adjusted based on our factor analysis findings and validated for use, we plan to utilize the survey to conduct a second analysis to assess the overall quality of our service and identify opportunities for program improvement within each domain. This will allow us to determine if the survey is helpful in gauging the impact of iterative quality improvement initiatives. Simultaneously, we will conduct additional consultations relating to new and emerging questions that arise in relation to patient experiences in telepsychiatry. Our team actively engages people with lived experience in the co-design of programs, and we will seek to involve patients in further improving the survey. Finally, we are always open to the feedback of health providers working in our program and those referring patients to our program. If changes are made as a result of these consultations, additional factor analysis and validation will be conducted.

After the advent of the COVID-19 pandemic, our team has also committed to taking a digital health equity perspective, working toward more equitable access to telehealth, while also recognizing that larger structural factors may impact access to technology and/or influence comfort with accessing care using technology [41]. In the future, we will ensure that the survey addresses equity, an important HSO identified by the Institute of Medicine [23]. We plan to include additional questions to assess equity as it relates to digital health, including questions on language, education, ethnicity, age, gender, and culturally safe and compassionate care.

In addition, qualitative research may help to illuminate some of the barriers and facilitators to the use of telepsychiatry by both patients and providers. Although measures of patient experience and satisfaction inform person-centered care, it is important to continue to be open to patient-derived expressions of experience and quality and to ensure that these are not overlooked.

### Limitations

Self-selection bias is a possible limitation to this study, as the ability to self-select may affect the response rate, which is unknown for this study. For example, the most satisfied or unsatisfied patients are more likely to respond to the survey [42]. To minimize nonresponse, telemedicine coordinators at the different community provider sites were asked to give the patient experience survey to all patients at the beginning of their appointment and to collect them upon completion. Another possible bias highlighted by Williams et al [43] is that telemedicine survey respondents may be more likely to respond favorably to satisfaction surveys given the perceived potential impacts on health care service delivery and access. It could be argued, however, that the perceived pressure to respond favorably is diminished in the case of telepsychiatry consultations, as in the context of our service, the consulting provider does not provide ongoing care to the patient, and patient responses are anonymous. In future research, it would be beneficial to know the response rate to better understand the magnitude of response bias. To account for the potential bias whereby patients rate all HSOs highly based on their preference for a particular psychiatrist [42], rather than the overall process or experience of their telepsychiatry appointment, we looked at aggregate means instead of data at the individual psychiatrist level.

Revising the model based partly on information from the data itself lowers the level of evidence for our final CFA model. Despite the fact that we only addressed the most relevant fit issues and based model modifications on theoretical considerations, it is possible that our final model is affected by overfitting problems. The only way to address that, however, would be through the replication of the analysis with a different data set, which we plan to do in future iterations.

### Conclusions

This study sought to address a notable gap in the literature with respect to validated measures of patient satisfaction with telepsychiatry. This study used HSOs as a guiding framework for the development and validation of a patient satisfaction and experience survey. By situating patient satisfaction and experience within this framework, the survey facilitates patient data collection and interpretation through a clinical quality lens. This study also illuminates the clinical quality domains that would benefit from targeted quality improvement initiatives to further improve overall patient experience and satisfaction with telepsychiatry.

## Abbreviations

CFA: confirmatory factor analysis
CFI: confirmatory fit index
EFA: exploratory factor analysis
HSO: health service outcome
RMSEA: root mean square error of approximation
WLSMV: weighted least squares with mean and variance-adjusted chi-square test statistic
